# Periodontitis and dental quality of life predict long-term survival in head and neck cancer

**DOI:** 10.1186/s12903-024-05170-0

**Published:** 2024-11-19

**Authors:** Mirna Farran, Evelyn Neppelberg, Sigbjørn Løes, Anne K. H. Aarstad, Svein Erik Moe, Hans Jørgen Aarstad

**Affiliations:** 1 Department of Oral and Maxillofacial Surgery, Haukeland University Hospital, Bergen, Norway; 2 Department of Clinical Dentistry, University of Bergen, Bergen, Norway; 3 Department of Otolaryngology, Head and Neck Surgery, Haukeland University Hospital, Bergen, 5021 Norway; 4 Faculty of Health Sciences, UiT - The Arctic University of Norway, Tromsø, Norway; 5 VID Specialized University, Bergen, Norway; 6 Department of Clinical Medicine, Faculty of Medicine, University of Bergen, Bergen, Norway

**Keywords:** Head and neck, Carcinoma, HPV, Oral health, Periodontitis, Health-related quality of life

## Abstract

**Background:**

Our aim was to investigate oral health in newly diagnosed head and neck squamous cell carcinoma (HNSCC) patients in relation to long-term survival. We assessed whether the level of alveolar bone loss due to periodontitis at diagnosis, measured from orthopantomogram (OPG), and reported dental health-related quality of life (HRQoL) scores obtained at diagnosis contain prognostic information for HNSCC patients.

**Methods:**

A total of 79 patients from a consecutive cohort of 106 diagnosed with HNSCC between November 2002 and June 2005 were included. All patients reported dental HRQoL, OPG-determined alveolar bone loss were measured in 79 patients at diagnosis. Reduced alveolar bone loss (≥ 4 mm) from cement-enamel junction on at least two molars or premolars registered both horizontally and vertically served as indicator of periodontal disease.

**Results:**

With alveolar bone loss, we determined increased mortality by univariate analysis (RR = 2.28, CI: 1.22–4.28, *p* = 0.01) and a strong trend by multivariate analyses adjusted for standard clinical information (RR = 1.95, CI: 0.98–3.87, *p* = 0.056). Reported lowered dental HRQoL scores predicted long-term survival in both univariate (RR = 3.58, CI: 1.99–6.45, *p* < 0.001) and multivariate adjusted for standard clinical information (RR = 2.17, CI: 1.17–4.01, *p* = 0.014). When analyzed with Cox regression, including alveolar bone loss and dental HRQoL, both factors, adjusted by clinical variables, were significant predictors of long-term survival: dental HRQoL (*p* = 0.007) and present alveolar bone loss (*p* = 0.034). Non-HNSCC disease-specific long-term survival predicted was also predicted when alveolar bone loss and dental HRQoL were analyzed simultaneously and adjusted for standard clinical information.

**Conclusions:**

The degree of alveolar bone loss, as determined by OPG, and dental HRQoL both obtained at the time of HNSCC diagnosis, predicted long-term survival. When analyzed simultaneously, both factors remained significant in both univariate and multivariate analyses, adjusted by pertinent clinical variables, highlighting their unique prognostic value.

## Background

Head and neck cancers (HNC), which include lip, oral cavity, pharynx, larynx and salivary glands, comprise nearly one million new cases worldwide, constituting about 5% of the worldwide cancer incidence according to Global Cancer Statistics [1, 2]. Despite advancements, only around 50% of newly diagnosed HNC patients worldwide achieve a cure within five years following diagnosis [1]. In Norway, HNC accounted for approximately 2.5% (*n* ≈ 800) of total cancer incidence, with curative treatment achieving five-year survival in about two-thirds of diagnosed patients [3].

Tobacco and alcohol, particularly in combination, are well-established as significant risk factors for HN squamous cell carcinoma (HNSCC) [4]. Specifically, in oropharyngeal SCC (OPSCC), human papillomavirus (HPV) is emerging as a crucial risk factor, with an increasing incidence of HPV-positive (HPV(+)) OPSCC in the Western world [5]. Patients with HPV(+) tumors exhibit a distinct biology from those with HPV-negative (HPV(−)) tumors, including difference in carcinogenesis [6]. HPV(+) OPSCC patients generally have a much better prognosis compared to their HPV(−) counterparts [7]. However, the prognosis for OPSCC HPV(+) deteriorates with increasing tobacco use [7]. Poor oral health is related to general mortality [8] and is also recognized as a significant risk factor for HNSCC [9].

Periodontal diseases [10] and caries [11] may serve as indicators of poor oral health [12] and are known risk factors related to survival in oral cancer [13]. Additionally, tobacco and alcohol consumption are established risk factors for both HNSCC and periodontal disease [14].

Health-related quality of life (HRQoL) scores, as determined by questions regarding oral and dental-related symptoms at the diagnosis of HNSCC, have demonstrated predictive value for survival both generally [15] and concerning HNSCC [16]. However, the underlying mechanisms behind these associations remain unclear. Several potential mechanisms exist, ranging from the influence of comorbidities [17] and health behaviors, such as tobacco consumption [18], to the impact of the oral microbiome [19].

In clinical HNSCC practice, assessing dental status is routine for newly diagnosed patients [20]. This evaluation primarily aims to prevent potential side effects of treatment, such as osteoradionecrosis following radiation therapy, and to plan for oral or dental reconstruction as necessary [20]. Emphasizing the importance of these assessments could help optimize patient outcomes [21].

In addition to HNSCC and other smoking-related carcinomas [9], periodontitis is associated with conditions such as diabetes, hypertension, lung disease, and Alzheimer’s disease, all of which are linked to increased mortality [22] Consequently, patients with extensive periodontitis *are expected to* have higher mortality compared to those with limited disease. Previous studies from our group have shown that periodontitis predicts non-disease-specific survival in patients with OPSCC [21]. In this study, we hypothesize that a similar relationship may exist with general HNSCC patients.

Orthopantomogram (OPG) imaging allows for standardized assessment of osseous lesions associated with periodontitis [23]. We aim to determine the prognostic value of present periodontitis in a cohort of HNSCC patients at the time of diagnosis. Specifically, we are interested in exploring whether survival predictions based on patient-reported dental health and the extent of periodontitis diagnosed via OPG overlap. Understanding the origin and implications of such survival predictions is a primary objective of this investigation.

Our study aims to assess both five-year and long-term survival predictions in a general HNSCC cohort, focusing on periodontal pathology measured from OPG at diagnosis and HRQoL scores obtained at the same time. Additionally, we will analyze results with and without including index HNSCC mortality to comprehensively evaluate these survival predictions.

## Methods

### Patients

Haukeland University Hospital, Bergen, Norway, treats HNC patients in the Western Health Care Region, which includes around 1.1 million inhabitants. Our hospital-based HNC register includes patients starting treatment since May 1, 1992. The present study is based on data from a consecutive cohort of 106 patients diagnosed from November 2002 to June 2005, all aimed at curative treatment. We required that the patients were able to answer HRQoL questionnaires intelligibly. The patient cut-off age at diagnosis was 78 years. The Regional Committee for Medical Research Ethics in Western Norway approved the study (2011/125). Informed consent to participate was obtained from all participants in the study.

All patients underwent standardized diagnostic work-up, which consisted of clinical examination, CT/MRI scans of the primary tumor site, neck, thorax, and liver, and ultrasonography examination of the neck including fine-needle aspiration cytology if indicated. Diagnostic endoscopic examinations (microlaryngoscopy, hypopharyngoscopy, bronchoscopy and esophagoscopy) were performed, preferably under general anesthesia if the patient was suitable. The TNM (Tumor, Nodes, Metastasis) stage was scored according to the International Union against Cancer (IUCC) 6th edition, which was the relevant standard at the time, although the 8th Edition is in use today [24]. The sites and TNM stages of patients are listed in Table 1.

As part of the routine pretreatment workup at our clinic for patients planned for radiation therapy (RT) to the oral cavity, the HNC patients underwent a dental screening examination in the Department of Oral and Maxillofacial Surgery. This examination consisted of a clinical and radiographic examination, including an OPG supplemented with dental radiographs if indicated.

In total, 106 patients were included. Of the original cohort, OPGs of 27 patients were not available. Of the patients without OPGs, 14 were not treated with RT and consequently did not undergo OPG. These patients included 10 with early-stage laryngeal cancer and four with early-stage oral cavity cancer. Additionally, five patients with laryngeal cancer were not subjected to OPG examination because the RT field did not reach the oral cavity. Thus, OPGs from eight patients were missing without explanation.

### Treatment

An overview of treatment performed is listed in Table 1. The patients’ treatment details have been reported in previous studies from our group [25].

Eighty patients underwent primary tumor surgery aimed at radically removing the tumor tissue when indicated. Intraoperative biopsies were taken from the margins for further characterization through frozen sections. Free flap surgery was performed on 21 patients. Neck dissection, following previously reported procedures [26], was conducted on 51 patients.

The radiation therapy (RT) administered is detailed in Table 1. RT was primarily given according to the Danish Head and Neck Cancer Group (DAHANCA) Guidelines, utilizing an external beam RT with a linear accelerator. The RT doses ranged from 64 to70 Gray for all macroscopic tumors with borders, and 50 Gy to the neck when pertinent risk but no clinical disease was present. Eighty-seven of 106 patients received radiation therapy, with 78 treated specifically with neck radiotherapy.

Eleven patients received chemotherapy as part of their primary HNSCC treatment (Table 1).

### Smoking level and alcohol consumption history

Patient cigarette smoking was recorded by noting the total years of smoking and estimating the mean level of cigarettes smoked per week. Alcohol consumption was determined by having patients select one of the following statements: never (1), less than 1 time per week (2), 1–2 times per week (3), previously more than 2 times per week (4), and presently more than 2 times per week (5).

### Health-related quality-of-life (HRQoL) inventories

The questionnaires were completed through a structured interview. HRQoL was determined by patients answering the validated Norwegian edition of the European Organization for Research and Treatment of Cancer (EORTC) Quality of life Questionnaire (QLQ) Head and Neck (H&N)-35 [27]. The QLQ H&N-35 comprises seven multi-item scales (pain, swallowing, senses, speech, social eating, social contact, and sexuality), and six symptom items (dental problems, opening mouth, dry mouth, sticky saliva, coughing, and feeling ill). The answers were given according to a 4-point Likert scale. These indices were transformed so that 100 points indicated maximum symptoms and 0 points indicated least symptoms. In this study, we have employed the questions about dental health.

### Comorbidities

Comorbidities were obtained using the validated chart-based Adult Comorbidity Evaluation (ACE)-27 scale measured at baseline [28]. The ACE-27 grades specific conditions into levels of severity: mild, moderate, or severe. Based on the highest-ranked single ailment, an overall comorbidity score (none, mild, moderate, or severe) was assigned. In cases where two or more moderate ailments registered in different disease entities, the overall comorbidity score was designated as severe.

### Periodontal status

Radiographic alveolar bone loss (ABL) was measured as the distance in millimeters (mm) from the cement-enamel junction or restoration margin to the alveolar bone crest at mesial and distal surfaces of molars and premolars. An indicator of periodontal pathology was registered if there was at least 4 mm of bone loss from the cement-enamel junction on at least two molars or premolars [23]. The measurements were adjusted according to the enlargement factor provided by the OPG (1.3). Additionally, distinctions between vertical and horizontal bone loss were noted. Other parameters recorded included the number of missing teeth, filled teeth, residual roots, dental care status, and the number of teeth with apical radiolucencies (Table 2).

The OPGs were uniformly acquired, and a single examiner scored the radiographic parameters using Sirona Sidexis software without knowledge of patient details. To assess methodological quality, 25 radiographs were randomly selected and scored by the same investigator on two different occasions at least four weeks apart. The examination showed less than 10% variability between two assessments for the same patient.

### DNA isolation and HPV DNA detection

Tumor samples were carefully reviewed by an expert pathologist to select representative tissue specimen. DNA was extracted from formalin-fixed, paraffin-embedded (FFPE) sections, which included both primary tumors tissues or lymph node metastatic lesions obtained during diagnostic or surgical procedures. Three 10 μm thick FFPE sections were first deparaffinized in xylene and ethanol. These sections were then digested overnight in ATL buffer and Proteinase K (Qiagen GmbH, Hilden, Germany) at 56 °C. Following digestion, DNA was extracted using the EZNA tissue DNA kit (Omega Bio-tek, Norcross, GA). The DNA concentration was measured with a NanoDrop spectrophotometer (Nanodrop, Minneapolis, MN). Detailed methods for HPV DNA detection have been previously published in our earlier works [7]. Briefly, for the detection of HPV DNA, standard Gp5+/Gp6 + primers were used. PCR was conducted with both positive and negative controls, and the PCR products were then separated on a 3% agarose gel. Only samples with distinct PCR bands were considered positive for HPV and were subsequently processed for HPV subtype identification through DNA sequencing. The PCR products were purified using the same primers as the initial PCR reaction. The HPV DNA sequences were identified using the NCBI BLAST Database.

### Statistics

Statistical analyses were performed using IBM SPSS Statistics for Windows, version 29 (IBM Corp, Armonk, NY, USA). A value of *p* < 0.05 was considered to indicate a statistically significant result. All p-values reported represent two-sided tests. Pearson correlation coefficients were used to assess correlation between variables. Analysis of variance (ANOVA) was performed to study differences between HPV-negative and HPV-positive patients. The associations between possible prognostic variables and survival were determined using the Kaplan–Meier estimator and Cox proportional hazards regression models. Survival rates are reported as pertinent percentage survival and/or relative risk (RR) with 95% confidence intervals (CI). Non-disease-specific survival is reported as overall survival with disease-specific survival subtracted.

## Results

### Clinical parameters and survival predictions

Clinical parameters are summarized in Table 1. The factors considered included the age of the patients at diagnosis, gender, HPV status, smoked years, mean cigarettes smoked per week, level of comorbidity (ACE-27), and clinical stage. These parameters were analyzed for their potential to predict long-term survival using univariate Cox regression analysis (Table 1). Patient survival data are updated as of July 31, 2023. The age of patients at diagnosis predicted subsequent survival with a relative risk (RR) of 1.04 per year (confidence interval (CI): 1.02–1.07, *p* = 0.002). Years smoked predicted survival, with an RR of 1.03 (CI: 1.01–1.05, *p* = 0.028). The same was observed for the number of cigarettes smoked per week, with an RR of 1.004 (CI: 1.00-1.007, *p* < 0.01). Comorbidity, as measured on the ACE-27 scale, was a significant predictor of survival with an RR of 1.4 (CI: 1.12–1.76, *p* = 0.004). Clinical stage also showed a predictive trend with an RR of 1.2 (CI: 1.00-1.45, *p* = 0.056). A trend was observed regarding HPV status and survival (RR = 0.58, CI: 0.32–1.05, *p* = 0.074) (Table 1). Data from the orthopantomogram (OPG) studies, detailing findings on alveolar bone loss and other relevant variables by HPV status, are shown in Table 2.

**Table 1:** Patient characteristics including socio-demographics, clinical information, treatment, and univariate long-term overall survival (OS)

Characteristics	HRQoL^a^ + OPG^b^	HRQoL^a^ only	Total	RR (95% CI)^c^	*p*-value
Age (years ± SD)	61±8	61±10	61±9	1.04 (1.02–1.07)	0.002*
Gender n(%)					n.s.
Female	11 (14%)	4 (15%)	15 (14%)		
Male	68 (86%)	23 (85%)	91 (86%)		
Education^d^ n(%)					n.s.
High school or less	52 (66%)	14 (52%)	66 (62%)		
College or more	27 (34%)	13 (48%)	40 (38%)		
Year smoked mean±SD	30±16	31±18	30±17	1.03 (1.01–1.05)	< 0.028*
Cigarettes per week mean±SD	42±55	41±70	42±59	1.004(1.00-1.01)	< 0.001*
ACE-27^e^ n(%)				1.40 (1.12–1.76)	0.004*
None	31 (39%)	13 (48%)	44 (42%)		
Mild	26 (33%)	8 (30%)	34 (32%)		
Moderate	14 (18%)	5 (18%)	19 (18%)		
Severe	8 (10%)	1 (4%)	9 (8%)		
Tumor site n(%)					n.a.
Laryngeal	11 (14%)	15 (56%)	26 (25%)		
Oral cavity	26 (33%)	7 (26%)	33 (31%)		
Oropharyngeal	25 (32%)	2(7%)	27 (25%)		
Others	17(21%)	3 (11%)	20 (19%)		
Clinical stage^f^ n(%)				1.20 (1.00-1.45)	0.056
I	7 (9%)	14 (52%)	21 (20%)		
II	11(14%)	5 (19%)	16 (15%)		
III	17 (22%)	2 (7%)	19 (18%)		
IV	44 (55%)	6 (22%)	50 (47%)		
HPV status n(%)				0.58 (0.32–1.05)	0.074
Tumor negative	59 (75%)	25 (93%)	84 (79%)		
Tumor positive	20 (25%)	2 (7%)	22 (21%)		
Treatment n(%)					n.a.
Tumor surgery	57 (72%)	23 (85%)	80 (75%)		
Neck dissection	38 (48%)	13 (48%)	51 (48%)		
Free flap reconstruction	19 (24%)	2 (7%)	21 (20%)		
Tumor radiotherapy	74 (93%)	13 (48%)	87 (82%)		
Neck radiotherapy	69 (87%)	9 (33%)	78 (74%)		
Chemotherapy	11(14%)	0 (0%)	11 (10%)		

* *p* < 0.05; n.s = not significant; n.a = not applicable

^a^ HRQoL = Health-Related Quality of Life

^b^ OPG = Orthopantomogram

^c^ Cox univariate analysis of long-term overall survival CI = Confidence interval; RR = Relative risk

^d^ Self-reported at diagnosis

^e^ Adult Comorbidity Evaluation scale-27

^f^ Clinical stage by 6th TNM edition

**Table 2:** Parameters from orthopantomogram (OPG) studies

Variable	Count level	HPV *n*(%)		*p*-value†
		(─)	(+)	(HPV(−) vs. HPV(+))
Vertical bone loss^a^	No	40 (68%)	15 (75%)	
	Yes	19 (32%)	5 (25%)	
Horizontal bone loss^a^	No	31(53%)	15 (75%)	
	Yes	28 (47%)	5 (25%)	
Apical radiolucency^b^	0	24 (41%)	10 (50%)	
	1	20 (34%)	6 (30%)	
	2	10 (17%)	3 (15%)	
	3+	5 (8%)	1 (5%)	
Missing teeth	0	7 (12%)	6 (30%)	*p* = 0.04*
	1–5	20 (34%)	8 (40%)	
	6–10	8 (14%)	4 (20%)	
	11–15	5 (8%)	0 (0%)	
	16–20	8 (14%)	0 (0%)	
	21+	11 (18%)	2 (10%)	
Filled teeth	0	6 (10%)	0 (0%)	
	1–5	9 (15%)	2 (10%)	
	6–10	7 (12%)	3 (15%)	
	11–15	12 (20%)	5 (25%)	
	16–20	19 (32%)	9 (45%)	
	21+	6 (10%)	1 (5%)	
Residual roots	0	47 (79%)	16 (80%)	
	1	8 (14%)	2 (10%)	
	2	1 (2%)	1 (5%)	
	3+	3 (5%)	1 (5%)	

†Statistics by Mann-Whitney U-test, *p < 0.05

^a^ Vertical or horizontal alveolar bone loss was measured radiographically as the distance in millimeters from the cement-enamel junction (or restoration margin) to the alveolar bone crest at the mesial and distal surfaces of molars and premolars. A bone loss of at least 4 mm from the cement-enamel junction on at least two teeth was registered as an indicator of periodontal pathology

^b^ Number of teeth with radiolucency at the tooth root’s apex

### Pearson correlations between variables

Age at diagnosis correlated negatively with HPV status (*r* = -0.26, *p* < 0.01) and positively with the level of comorbidity measured by ACE-27 (*r* = 0.23, *p* < 0.05) (Table 3). HPV status correlated positively with clinical stage (*r* = 0.35, *p* < 0.001), and negatively with years smoked (*r* = -0.36, *p* < 0.001) and number of cigarettes smoked per week (*r* = -0.25, *p* < 0.05). Years smoking correlated with both reported dental HRQoL (*r* = 0.20, *p* < 0.05) and the level of alveolar bone loss (*r* = 0.39, *p* < 0.001). The level of alveolar bone loss also correlated with the comorbidity level measured by ACE-27 (*r* = 0.36, *p* < 0.001) (Table 3).

**Table 3:** Pearson correlations between clinical and study variables

	Age	Gender	HPV status	Clinical stage	ACE-27	Years smoking	Cigarettes per week	Alcohol history	Dental HRQoL
Gender	0.04								
HPV status	− 0.26**	− 0.06							
Clinical stage	0.02	0.08	0.35***						
ACE-27	0.23*	0.14	− 0.09	− 0.05					
Years smoked	0.28**	0.25*	− 0.36***	-0.1	0.29**				
Cigarettes per week	− 0.07	0.04	− 0.25*	− 0.11	0.15	0.48***			
Alcohol history	− 0.06	0.09	− 0.05	− 0.04	0.05	0.28**	0.21*		
Dental HRQoL	0.14	0.12	0.06	0.20*	0.13	0.20*	0.22*	0.02	
Alveolar bone loss	0.14	0.16	− 0.02	− 0.26*	0.36***	0.39***	0.14	− 0.00	0.05

* *p* < 0.05 **p < 0.01 *** *p* < 0.001

### Five-year survival

**Fig. 1:**
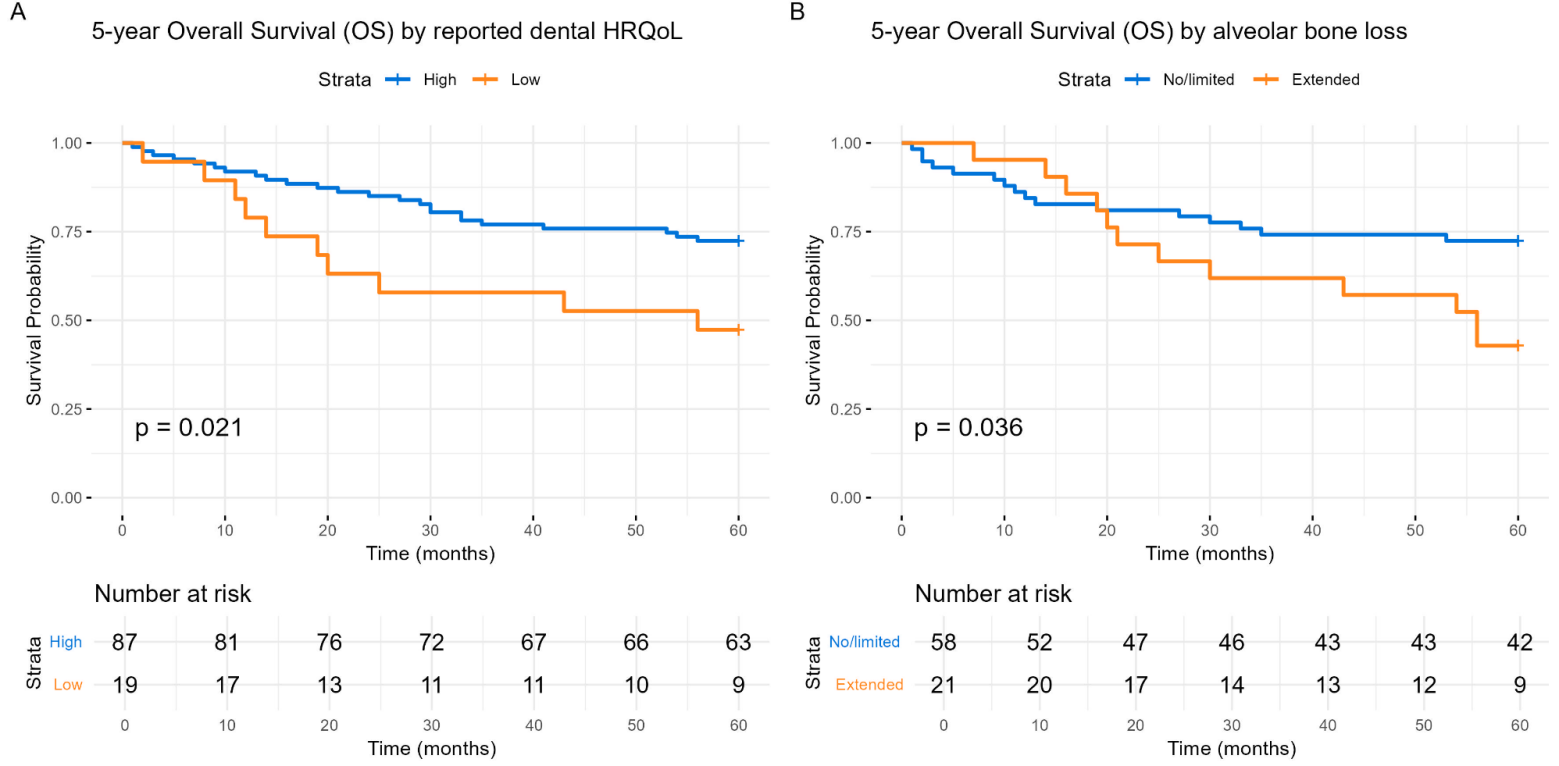
Kaplan-Meier 5-year overall survival dependent on reported dental HRQoL and the presence of alveolar bone loss. (**A**) 5-year overall survival of HNSCC patients based on reported dental HRQoL. Dental HRQoL was assessed using the EORTC H&N35 questionnaire answered at diagnosis. High reported dental HRQoL is shown in blue, and low dental HRQoL is shown in orange. (**B**) 5-year overall survival of HNSCC patients based on the presence of alveolar bone loss. Alveolar bone loss was determined from OPGs at diagnosis, with measurements indicating reduced alveolar bone loss (≥ 4 mm) from the cement-enamel junction on at least two teeth both horizontally and vertically, defined as extended periodontitis. No/limited alveolar bone loss is shown in blue, and extended alveolar bone loss is shown in orange. The Y-axis represents the probability of survival, and the X-axis denotes time in months. Differences between the groups are examined with log-rank tests and presented with p-values

**Fig. 2:**
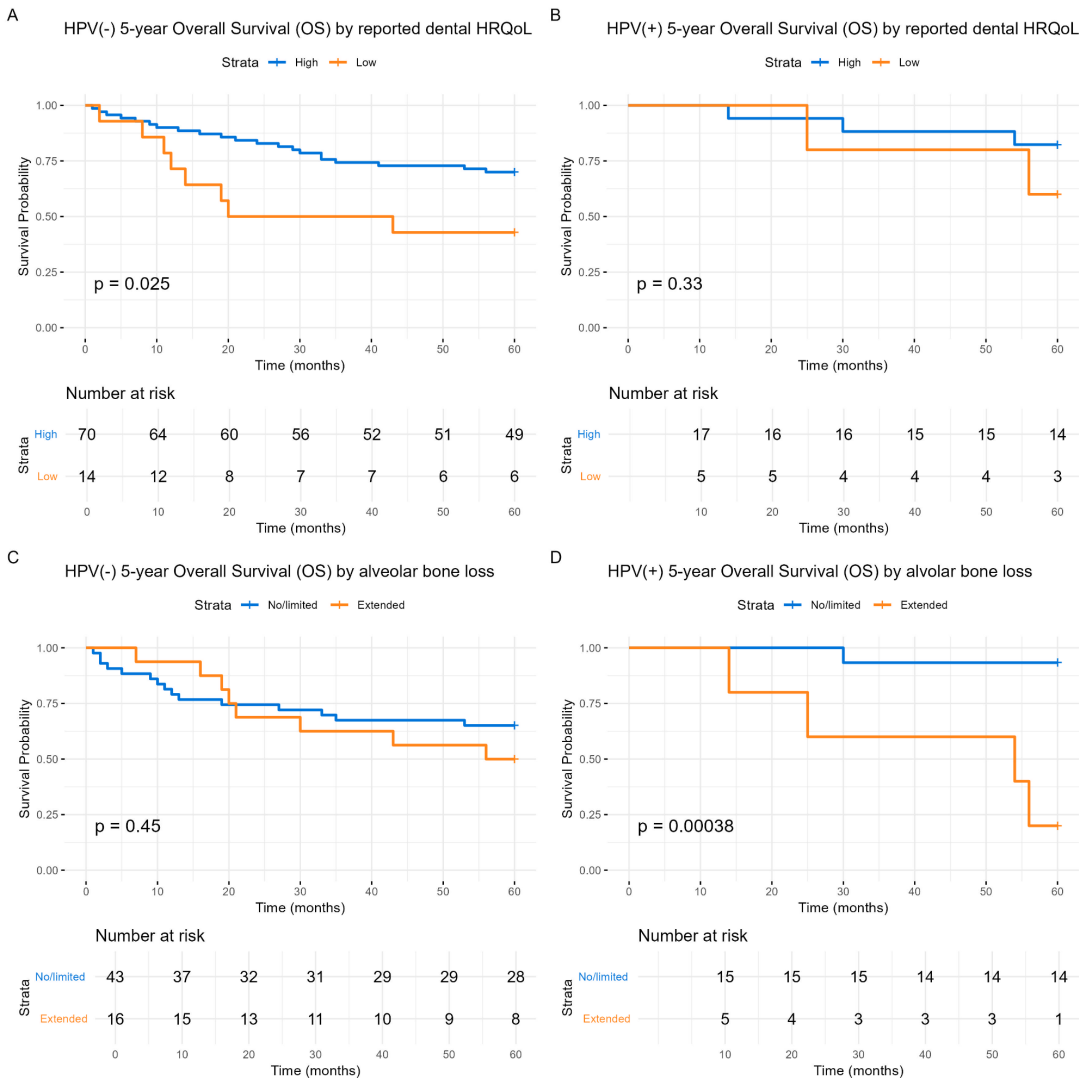
Kaplan-Meier 5-year overall survival dependent on reported dental HRQoL and the presence of alveolar bone loss, stratified by HPV status. (**A**) HPV(−): 5-year overall survival by reported dental HRQoL. (**B**) HPV(+): 5-year overall survival by reported dental HRQoL. High reported dental HRQoL is shown in blue, and low dental HRQoL is shown in orange. (**C**) HPV(−): 5-year overall survival by alveolar bone loss. (**D**) HPV(+): 5-year overall survival by alveolar bone loss. No/limited alveolar bone loss is shown in blue, and extended alveolar bone loss is shown in orange. The Y-axis represents the probability of survival, and the X-axis denotes time in months. Differences between the groups are examined with log-rank tests and presented with p-values

Five-year survival of the studied cohort by patient-reported dental HRQoL or alveolar bone loss are demonstrated in Fig. 1. Both entities, i.e., dental HRQoL (RR = 2.85, CI: 1.17–7.01, *p* = 0.021) and alveolar bone loss (RR = 2.80, CI: 1.04–7.53, *p* = 0.036), predicted survival (Fig. 1). When stratified by tumor HPV status (Fig. 2), the dental HRQoL prediction was mainly significant among HPV(−) patients (*p* = 0.025) (Fig. 2A), whereas alveolar bone loss prediction was more pronounced among HPV(+) patients (*p* < 0.001) (Fig. 2D).

In a Cox multivariate regression model including variables measured at diagnosis (age of patient, gender, HPV status, clinical stage, smoking and alcohol history, and comorbidity (ACE-27)), dental HRQoL predicted survival (RR = 2.53, CI: 1.02–6.24, *p* = 0.045) (Table 4). When both dental HRQoL and alveolar bone loss were included in the same Cox multivariate analysis alongside the covariates mentioned above, dental HRQoL remained a significant predictor of survival (*p* = 0.037), while rate of alveolar bone loss showed a trend towards predicting survival, but it did not reach statistical significance (*p* = 0.076) (Table 4).

**Table 4:** 5- year overall survival studied by multivariate Cox regression analyses. Clinical variables are included in block I, as well as reported dental HRQoL and alveolar bone loss

5-year survival	*p*-value	RR	95% CI for RR	
			Lower	Upper
**Block I**				
Age at diagnosis	0.417	0.98	0.93	1.03
Gender	0.340	2.03	0.47	8.71
HPV status	0.311	0.56	0.19	1.71
Clinical stage	0.016*	1.54	1.09	2.19
Cigarettes per week	0.089	0.99	0.98	1.00
Years smoked	0.060	1.03	0.99	1.07
Alcohol consump-tion history	0.139	1.27	0.93	1.75
ACE-27	0.032*	1.49	1.04	2.16
**Block I + dental HRQoL**				
Dental HRQoL	0.045*	2.53	1.02	6.24
**Block I + alveolar bone loss**				
Alveolar bone loss	0.122	2.09	0.82	5.29
**Block I + dental HRQoL and alveolar bone loss together**				
Dental HRQoL	0.037*	2.98	1.07	8.34
Alveolar bone loss	0.076	2.46	0.91	6.61

* *p* < 0.05

### Long-term survival: 18–20 years

**Fig. 3:**
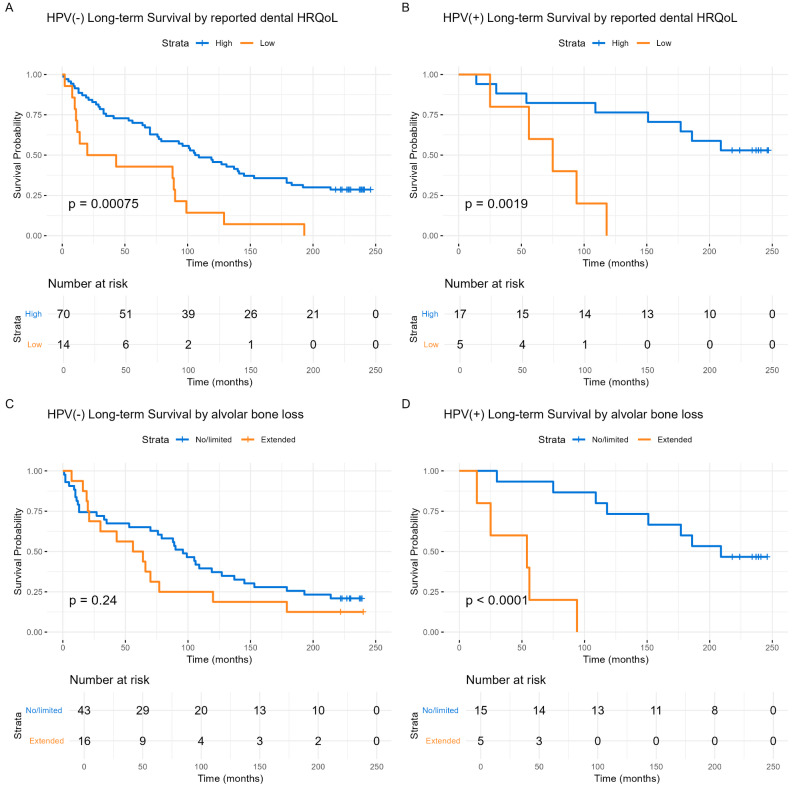
Kaplan-Meier long-term overall survival dependent on reported dental HRQoL and presence of alveolar bone loss, stratified by HPV status. (**A**) HPV(−): Long-term overall survival by reported dental HRQoL. (**B**) HPV(+): Long-term overall survival by reported dental HRQoL. High reported dental HRQoL is shown in blue, and low dental HRQoL is shown in orange. (**C**) HPV(−): Long-term overall survival by alveolar bone loss. (**D**) HPV(+): Long-term overall survival by alveolar bone loss. No/limited alveolar bone loss is shown in blue, and extended alveolar bone loss is shown in orange. The Y-axis represents the probability of survival, and the X-axis denotes time in months. Differences between the groups are examined with log-rank tests and presented with p-values

Perceived dental HRQoL also predicted long-term survival (RR = 3.58, CI: 1.99–6.45, *p* < 0.001). When adjusted for HPV status, this association was maintained for both HPV(−) (*p* < 0.001) and HPV(+) (*p* = 0.002) patients (Fig. 3A).

Alveolar bone loss also predicted survival (RR = 2.28, CI: 1.22–4.28, *p* = 0.01). When stratified by HPV status, survival was predicted by alveolar bone loss among HPV(+) patients (*p* < 0.001) (Fig. 3D).

Long-term survival was further studied using Cox regression multivariate analyses (Table 5). Control covariates included age at diagnosis, gender, HPV tumor status, clinical stage, smoking, alcohol use, and comorbidity (ACE-27). In this analysis, reported dental HRQoL predicted survival (RR = 2.17, CI: 1.17–4.01, *p* = 0.014), while the rate of alveolar bone loss showed a trend toward predicting survival but did not reach statistical significance (RR = 1.95, CI: 0.98–3.87, *p* = 0.056). When both dental HRQoL and alveolar bone loss were included in a single regression analysis with the above-mentioned covariates, significant unique survival predictions were obtained both dental HRQoL (*p* = 0.007) and alveolar bone loss (*p* = 0.034) (Table 5).

**Fig. 4:**
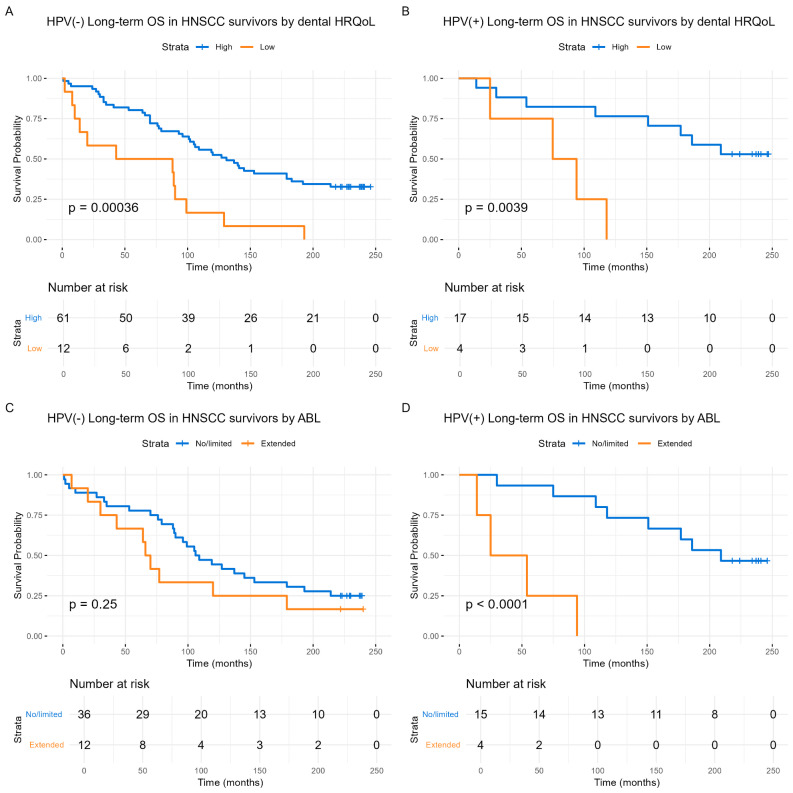
Kaplan-Meier long-term survival among index HNSCC disease-specific survivors, dependent on reported dental HRQoL and the presence of alveolar bone loss, stratified by HPV status (**A**) HPV(−): Long-term overall survival among HNSCC disease-specific survivors by reported dental HRQoL. (**B**) HPV(+): Long-term overall survival among HNSCC-disease-specific survivors by reported dental HRQoL. High reported dental HRQoL in blue, and low dental HRQoL is shown in orange. (**C**) HPV(−): Long-term overall survival among HNSCC-disease-specific survivors by alveolar bone loss. (**D**) HPV(+): Long-term overall survival among HNSCC disease-specific survivors by alveolar bone loss. No/limited alveolar bone loss is shown in blue, and extended alveolar bone loss is shown in orange The Y-axis represents the probability of survival and, and the X-axis represents time in months. Differences between groups are examined with the log-rank tests and presented with p-values

Long-term survival was also analyzed by using Kaplan-Meier methods, focusing on patients who survived the HNSCC index disease. Reported dental HRQoL predicted survival (RR = 3.58, CI: 1.99–6.45, *p* < 0.001). Stratifying by HPV tumor status, significant survival predictions were observed for HPV(−) (*p* < 0.001) and HPV(+) (*p* = 0.004) groups (Fig. 4A and B). Alveolar bone loss, including all surviving patients, also predicted survival (RR = 2.28, CI: 1.22–7.78, *p* = 0.010). When stratified by HPV status, significant survival predictions was observed only among HPV(+) patient (*p* < 0.001) (Fig. 4D).

Long-term survival among HNSCC disease-specific survivors was also assessed using Cox regression analyses using the aforementioned covariates (Table 6). This analysis demonstrated that reported dental HRQoL (RR = 2.76, CI: 1.24–6.15, *p* = 0.013) and the extent of alveolar bone loss (RR = 2.66, CI: 1.18–5.96, *p* = 0.018) independently predicted survival when analyzed concurrently (Table 6).

**Fig. 5:**
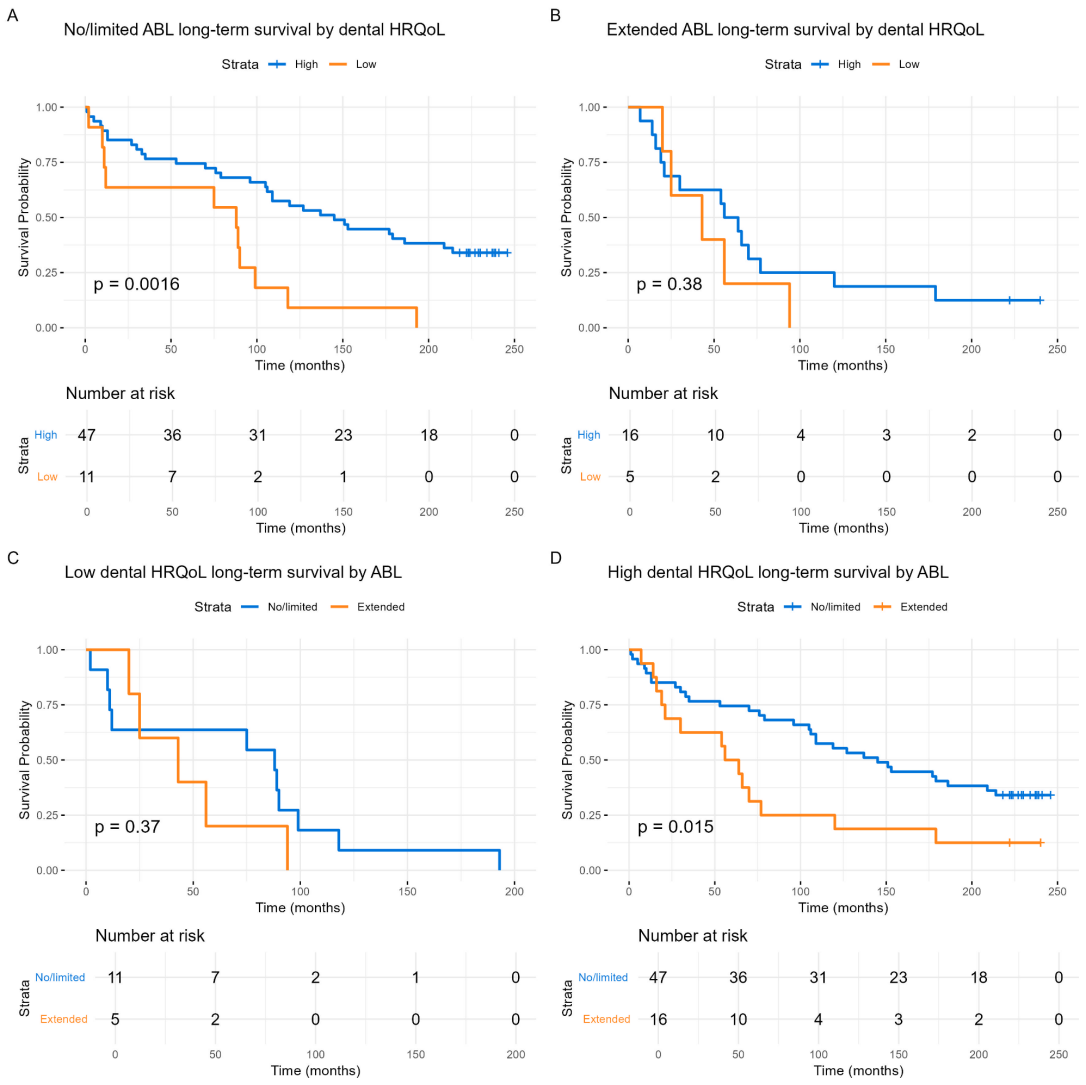
Long-term survival of HNSCC patients by reported dental HRQoL adjusted by alveolar bone loss, and by alveolar bone loss adjusted by reported dental HRQoL. (**A**) No/limited alveolar bone loss. (**B**) Extended alveolar bone loss. High reported dental HRQoL is shown in blue, and low dental HRQoL is shown in orange. (**C**) Low reported dental HRQoL (**D**) High reported dental HRQoL. No/limited alveolar bone loss is shown in blue, and extended alveolar bone loss is shown in orange. The Y-axis represents the probability of survival, and the X-axis denotes time in months. Differences between groups are examined with log-rank tests and presented with p-values. Statistics: *p* < 0.001 combined

Finally, we investigated whether the reported level of dental HRQoL, adjusted by alveolar bone loss, continued to predict survival, which indeed was the case (*p* < 0.001) (Fig. 5A). Similarly, the reverse analysis also showed significance (*p* = 0.017) (Fig. 5D).

**Table 5:** Long-term overall survival by multivariate Cox regression analyses including clinical variables (block I), reported dental HRQoL and alveolar bone loss

Long-term survival	*p*-value	RR	95 CI for RR	
			Lower	Upper
**Block I**				
Age at diagnosis	0.037*	1.04	1.00	1.07
Gender	0.242	1.56	0.74	3.27
HPV status	0.416	0.74	0.35	1.54
Clinical stage	0.005*	1.35	1.10	1.66
Cigarettes per week	0.284	1.00	0.99	1.01
Years smoking	0.174	1.01	0.99	1.03
Alcohol history	0.340	1.11	0.90	1.37
ACE-27	0.063	1.25	0.99	1.59
**Block I + dental HRQoL**				
Dental HRQoL	0.014*	2.17	1.17	4.01
**Block I + alveolar bone loss**				
Alveolar bone loss	0.056	1.95	0.98	3.87
**Block I + dental HRQoL + alveolar bone loss**				
Dental HRQoL	0.007*	2.62	1.31	5.28
Alveolar bone loss	0.034*	2.13	1.06	4.28

* *p* < 0.05

**Table 6:** Long-term overall survival by multivariate Cox regression analysis including clinical variables, dental HRQoL and alveolar bone loss studying only index disease-survived patients

	*p*-value	RR	95% CI for RR	
			Lower	Upper
Age at diagnosis	0.033*	1.05	1.00	1.09
Gender	0.025*	3.13	1.16	8.47
HPV status	0.861	1.08	0.45	2.58
Clinical stage	0.114	1.32	0.94	1.87
Cigarettes per week	0.860	0.99	0.99	1.01
Years smoked	0.346	1.01	0.10	1.04
Alcohol history	0.234	1.18	0.90	1.56
ACE-27	0.042*	1.36	1.01	1.84
Dental HRQoL	0.013*	2.76	1.24	6.15
Alveolar bone loss	0.018*	2.66	1.18	5.96

* *p* < 0.05

## Discussion

From HNSCC patients, we determined the extent of alveolar pathology based on blind scoring from routine orthopantomograms (OPGs) and patient-reported dental HRQoL, both obtained during the primary diagnostic work-up (see Table 1). The results showed that present alveolar bone loss (Figs. 1, 2 and 3), together with low patient reported dental HRQoL (Figs. 1, 2 and 3), uniquely predicted decreased long-term survival (Fig. 5; Tables 4 and 5). Furthermore, non-HNSSC disease-specific long-term survival was also predicted (Fig. 4; Table 6).

The assessment of periodontal alveolar bone loss was based on OPGs. Several measures derived from an OPG are presented in Table 2. Previously, studying periodontal bone loss has proved a useful approach regarding survival both generally [29] and regarding HNSCC patients [21, 30]. Studying horizontal and alveolar vertical bone loss separately has been employed. The best survival prediction was shown to be the sum of the horizontal and vertical bone loss [21], and this has therefore mainly been used in this study.

Ideally, results from clinical investigations of the level of marginal periodontitis could also have been included. The accuracy of estimating the degree of marginal periodontitis by clinical examinations compared to OPG been investigated by Bueno et al. [23]. They showed that defining marginal periodontitis as ≥ 2 sites with interproximal clinical attachment loss ≥ 4 mm in alveoli from at least two different teeth as seen on an OPG, was comparable to defining marginal periodontitis clinically [23]. This is the presently used measure.

Furthermore, an advantage has been that the OPGs were scored by a single investigator who had no separate clinical knowledge about the patients. Therefore, data acquisition can be considered blind and to some extent prospective. We have previously shown that a high degree of periodontal bone loss in newly diagnosed patients with oropharyngeal carcinoma may predict lowered subsequent survival [21]. Currently, this prediction is being validated in a general cohort of newly diagnosed HNSCC patients (Figs. 1, 2, 3 and 4). This may help to establish personalized treatment by identifying patients with a serious prognosis.

The questions regarding dental HRQoL were sampled from the EORTC QoL H&N specific part [28]. We, along with others, have previously shown that the response pattern to this questionnaire can predict subsequent survival, both generally [15] and specifically for HNSCC [31]. This survival prediction has now been validated for both short-term (Fig. 2) and long-term survival (Fig. 3), as well as among the HNSCC survivors (Fig. 4). It suggests that this method can be used to identify patients who should be offered close follow-up.

One aim of this investigation was to determine to what extent survival predictions from alveolar bone loss and reported dental HRQoL overlap. The survival predictions from these entities were statistically independent of each other (Tables 4, 5 and 6; Fig. 5). The findings furthermore suggests a general survival pattern, with periodontal disease interacting differently with tumor HPV(+) versus HPV(−) HNSCC patients (Figs. 2 and 3). The HRQoL survival prediction is more important among HPV(−) patients, while alveolar bone loss is more important among HPV(+) patients.

Presently, comorbidity and smoking were related to periodontal status as reported in Table 3 [32]. The study design, however, does not allow establishing the cause of death except for the index HNSCC. It is likely that much of the non-HNSCC specific deaths observed may be due to smoking-related cancer and smoking-related cardiovascular disease, both also being associated with periodontal disease [33]. We have shown that both information about smoking history and the presence of comorbidity at least to some extent may serve as covariates without loss of the periodontal survival predictions (Tables 4, 5 and 6).

Patients with extensive alcohol consumption typically show a poor degree of maintaining their dental health [34], with alcohol consumption also being a risk factor for HNSCC [32]. As s far as studied, we can conclude that the present basic findings are not secondary to the alcohol consumption rate.

This work is based on studying minimum 77 patients. Multivariate Cox regression analyses with many introduced covariates, as in this case, can only suggest relationships between these covariates and survival predictions (Tables 4, 5 and 6). Therefore, this study should primarily inspire other investigators to include dental/oral HRQoL and periodontal level scores in their research to further detail the interactions between the studied covariates.

The suggested uniform criteria for defining and measuring the extent or severity of periodontitis primarily rely on clinical examinations [35]. However, information from OPGs can also be utilized, as this type of examination is easy to standardize and realistically to perform within the short time frame between cancer diagnosis and start of treatment. Additionally, asking patients to complete a HRQoL questionnaire is straightforward. Therefore, this study highlights the use of readily available variables as basis for personalized medicine. Consequently, information on treatment choice and especially individualized follow-up may be individually recommended.

Periodontitis is furthermore a chronic inflammatory disease characterized by progressive loss of alveolar bone and periodontal attachment [35]. Microbiota in dental biofilm and their harmful products trigger a host immune response, which in susceptible individuals may lead to destruction of periodontal tissue [36]. Periodontitis has been associated with various systemic diseases, and shared inflammatory pathways have been proposed as a possible explanation [10]. Inflammatory cytokines, soluble cytokine inhibitors, and/or soluble cytokine receptors may also provide a communication channel through which periodontitis can increase the risk of conditions such as cancer [37]. The opportunistic pathogen Fusobacterium nucleatum, which acts as a bridge between early and late colonizers in the dental biofilm [38], is among the periodontal bacteria frequently mentioned in connection with cancer progression and prognosis [39, 40], possibly through inflammatory pathways. The present results may support this suggestion. It would be of interest to further study the association between periodontitis and inflammatory activation in relation to patient prognosis [41].

Studies have shown systematic effects of periodontal disease treatment on conditions like diabetes [42]. In line with this, treatment of periodontitis, which leads to less inflammation and fewer oral pathogens, could be systematically studied through formal phase II-III trials, in HNSCC patients, with the main aim of preventing mortality. Such studies could also improve HRQoL for the patients, providing another mechanism for disease mitigation.

## Conclusion

The present work has demonstrated that periodontitis at the time of HNC diagnosis predicts subsequent survival. Similarly, patients reporting low dental HRQoL experienced worse survival outcomes. This information may serve as a basis for treatment decisions. However, many questions about these findings remain unanswered, and further studies are needed to explore the relationship between HNC, dental HRQoL, and periodontitis, ideally in formal phase II/III study settings.

## Data Availability

The data that support the findings of this study are available from the corresponding author upon reasonable request.

## Abbreviations

ABL: Alveolar bone loss
ACE: Adult Comorbidity Evaluation
ANOVA: Analysis of variance
CI: Confidence interval
FFPE: Formalin-fixed, paraffin-embedded
HNC: Head and neck cancer
HNSCC: Head and neck squamous cell carcinoma
HRQoL: Health-related quality of life
HPV: Human papillomavirus
HPV(−): Human papillomavirus-negative
HPV(+): Human papillomavirus-positive
OPG: Orthopantomogram
OPSCC: Oropharyngeal squamous cell carcinoma
RR: Relative risk
RT: Radiation therapy
TNM: Tumor, Nodes, Metastasis
